# What’s gut got to do with it? The role of the microbiota and inflammation in the development of adiposity and obesity

**DOI:** 10.1097/IN9.0000000000000029

**Published:** 2023-07-24

**Authors:** Travis Jennings, Mallory Janquart, Catherine Washak, Khrystyne Duddleston, Courtney Kurtz

**Affiliations:** 1 Department of Biological Sciences, University of Alaska Anchorage, Anchorage, AK, USA; 2 Department of Biology, University of Wisconsin Oshkosh, Oshkosh, WI, USA

**Keywords:** gut microbiota, white adipose tissue, liver, skeletal muscle, cytokines

## Abstract

Obesity is a complex and heterogeneous disease characterized by increased adiposity, ie, the accumulation of lipids and the growth of adipose tissue. In this mini-review, we explore the important role of the gut microbiota and immune system in the development of adiposity. Dysbiosis of the microbiota leads to increased permeability of the gut barrier and bacterial products in the bloodstream, which triggers metabolic inflammation of adipose tissue, muscle, and liver. Inflammation in these highly metabolic organs exacerbates adiposity and contributes to the development of comorbidities associated with obesity. Studies in animal models that manipulate the microbiota and/or inflammation have shown promise in the treatment of obesity.

## 1. Introduction

Obesity is a significant health problem affecting millions of people across the globe. The main diagnostic marker of obesity is an increase in the body mass index (BMI), a calculation involving weight and mass. The increased BMI in obese individuals is caused by an increase in white adipose tissue (WAT) due to hypertrophy and hyperplasia of this important energy-storage organ. This increased adiposity can contribute to several comorbidities, including heart disease, cancer, and type II diabetes mellitus (T2DM).

Obesity is thought to begin with abnormal regulation of satiety and hunger signals to the brain from peripheral tissues (eg, gut, liver and adipose) or within the brain itself ^[1]^. This dysregulation leads to changes in eating habits, energy metabolism, and/or autonomic function. Increased consumption of high-fat foods, in particular, tends to lead to metabolic inflammation and insulin resistance (IR) ^[2,3]^. Not surprisingly, these changes to eating habits affect homeostasis in the gastrointestinal tract (ie, the gut), where a delicate balance exists between a large population of commensal microorganisms and the largest immune cell population of the body. Separating these two groups is a single layer of epithelial cells. A significant disruption of the microbial community, like that seen in obesity, is enough to disrupt this system, leading to the activation of the gut immune cells and subsequent systemic inflammation. In this invited review, we summarize the basic pathway proposed to explain how a change of diet and disruption of the gut microbial community leads to inflammation of metabolic organs and the pathophysiology of obesity.

## 2. Gut microbiota and adiposity

The gut plays host to a dynamic community of commensal microorganisms with numbers in the trillions. This diverse microbiome plays a crucial role in human health by modulating several physiological functions and maintaining gut homeostasis ^[4]^. The intestinal mucosa is a semipermeable barrier responsible for nutrient absorption from the dietary products within the lumen and preventing pathogens and toxins in the lumen from entering the bloodstream ^[5]^. There are four key components to this complex system—the gut microbiota, the mucus layer, the epithelial monolayer, and the lamina propria ^[5]^. The last two components house large populations of host immune cells. The host’s ability to resist infection by pathogens requires strict regulation of this system maintained via the interaction between the host immune system and the microbiome ^[6]^. In this way, the immune system shapes and regulates the gut microbiota to keep a symbiotic relationship between residents and the host ^[7]^. Disease and other threats to homeostasis arise when this relationship becomes unbalanced (ie, dysbiosis).

Maintenance of an intact and robust gut barrier is essential to proper function and overall health. When the gut microbiome is in a state of dysbiosis, the translocation of bacterial products across the epithelial layer can lead to metabolic endotoxemia resulting from increased intestinal permeability ^[7,8]^. This permeability is thought to be a key driver in chronic inflammation due to the translocation of bacterial products such as lipopolysaccharide (LPS), which triggers the host immune response ^[9]^. Certain microorganisms, such as the mucin-degrading bacterium *Akkermansia muciniphila (A. muciniphila*), strengthen the barrier through interactions with host cells. *A. muciniphila* utilizes intestinal epithelial mucins as its major carbon and nitrogen sources, degrading the highly glycosylated proteins to acetate and propionate as metabolic end-products ^[10]^. These metabolites, known as short-chain fatty acids, are taken up by enterocytes and serve as energy sources for host epithelial cell turnover, improve the integrity of the epithelial cell layer, and assist the regulation of gut barrier function ^[11,12]^. Some members of the gut microbiota secrete spherical lipid bilayer structures known as extracellular vesicles (EVs) ^[8,13]^ that comprise proteins, lipids, nucleic acids, LPS, and other virulence factors and are thought to be conduits for the transfer of genetic material and proteins from bacteria to host ^[13,14]^. EVs interact directly with the gut epithelium and host immune cells to generate signaling pathways protective to the host ^[15]^. In essence, EVs are the functional units of the gut microbiota responsible for regulating host-pathogen interactions ^[8]^. For example, *A. muciniphila*-derived EV (AmEV) treatment increased tight junction protein expression in Caco-2 cells and improved intestinal barrier integrity in high-fat diet (HFD)-induced diabetic mice ^[8]^. Additionally, the number of AmEVs was higher in the feces of healthy control mice compared with diabetic mice, and supplementation with AmEVs reduced weight gain and improved glucose tolerance ^[8]^. These studies demonstrate the importance of certain members of the microbiota, such as *A. muciniphila* in maintaining gut barrier integrity and suggest that the use of these organisms as probiotics may be a viable treatment for metabolic inflammation caused by obesity.

**Figure 1.**
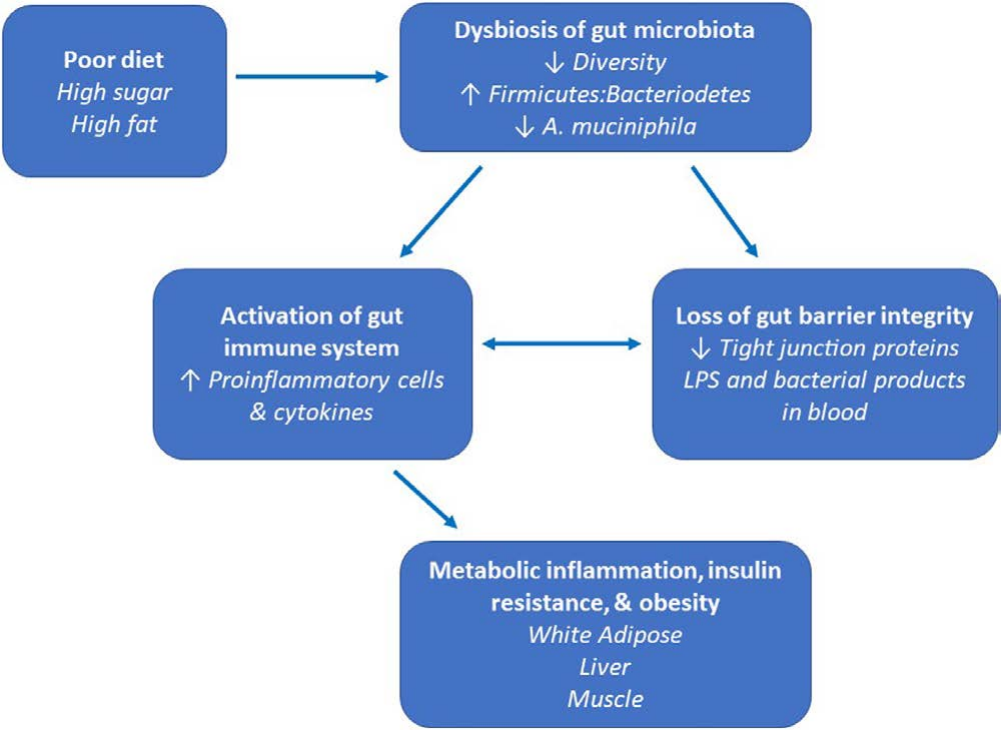
**The pathway to metabolic inflammation begins with a change in diet, leading to dysbiosis of the gut microbiota**. Dysbiosis decreases gut barrier integrity and leads to intestinal inflammation. This inflammation, coupled with the presence of microbial products in the bloodstream activates inflammatory pathways in distant metabolic organs like white adipose tissue (WAT), liver, and skeletal muscle.

Changes to the composition of the gut microbial community are often an indicator of disease, such as obesity. While the definition of what constitutes a healthy gut microbiome remains undefined, the gut microbiota of healthy (nonobese) individuals is highly diverse ^[16]^. Obesity is often associated with the loss of alpha diversity, the microbial diversity within a functional community, such as the gut microbiome at a given point in time ^[17,18]^ (Figure 1). An HFD has also been associated with phylum-level changes in gut microbiota involving the two most dominant phyla, Firmicutes and Bacteroidetes ^[19]^. HFD leads to an increased ratio of Firmicutes (known for their ability to metabolize dietary fiber) to Bacteroidetes ^[17,20]^. The expansion of the relative abundance of Firmicutes at the expense of Bacteroidetes is thought to result in an increased energy harvest and excess energy storage in adipose ^[17,19]^. Prolonged exposure to HFD directly correlates with an increase in *Bacillaceae*, *Clostridiaceae*, and other Firmicutes in the gut of obese mice, along with a decrease in *Bacteroides* and other Bacteroidetes ^[21]^. In addition, HFD leads to an overgrowth of *Oscillibacter* spp. (a Firmicute) whose presence triggers the release of proinflammatory cytokines from immune cells ^[22]^. However, to say that all Firmicutes induce an obese state would be misleading. For example, *Lactobacillus acidophilus*, a member of the phylum Firmicutes, improved body and adipose mass, glucose metabolism, and gut barrier function in an HFD model of obesity ^[23]^. Other bacterial phyla also play a role in the maintenance of gut homeostasis, including Verrucomicrobia (to which *A. muciniphila* belongs) and Actinobacteria. Supplementation with *Bifidobacterium* sp., a popular probiotic, and member of the Actinobacteria phylum, improves outcomes in obesity models through effects on gut barrier function and inflammation ^[24,25]^. Together, these studies demonstrate how small changes to the microbiota community can have dramatic effects on adiposity.

## 3. Gut inflammation and adiposity

Proper host-microbial interactions are essential for a healthy gut. These interactions begin with the intestinal epithelium, a single layer of columnar epithelial cells that separates the massive microbial community from a large immune population. Disruption of this delicate system, such as the dysbiosis mentioned earlier, can lead to loss of gut barrier integrity, endotoxemia (from LPS), and systemic inflammation. Key components of the gut barrier include tight junction proteins, namely zonula occludens-1 (ZO-1) and occludin, antimicrobial peptide-secreting Paneth cells, mucin-secreting goblet cells, and secretory immunoglobulin A ^[26]^. The intestinal epithelium is intended to be a continuous barrier to prevent the translocation of bacterial products from the lumen to the bloodstream, but this barrier can be disturbed by obesity and ingestion of HFD ^[27]^ due, at least in part, to decreased expression of ZO-1 and occludin ^[24]^ (Figure 1). Beyond molecular disruption of the gut barrier, HFD can lead to physical changes in the gut. Consumption of HFD decreased colon length, cecal weight, cecal and ileal crypt depth, and the number of goblet cells per crypt in rats, a phenomenon that can affect fermentation, nutrient absorption, and potential signals from the gut to the brain ^[27]^. Once intestinal permeability is increased, or conversely, barrier function is decreased, LPS and other bacterial products can pass through the barrier into the bloodstream ^[27]^. An increased concentration of LPS-binding protein (and therefore LPS) in circulation has been reported in obese human patients ^[26]^. In obesity, the presence of LPS in the bloodstream leads to immune signaling and a persistent low-grade inflammatory state. Furthermore, a large population of immune cells resides in the gut itself and can respond to these changes.

The gut immune population is normally tolerant of microbiota antigens, but when triggered by dysbiosis, can activate inflammatory pathways. Host epithelial and resident immune cells are continuously surveying bacterial cells along the mucosal surface via toll-like receptors (TLRs). These highly conserved receptors detect pathogen-associated molecular patterns, such as LPS, and subsequently activate an appropriate immune response ^[28]^. Homeostasis in the intestine is kept balanced by interactions between TLR4 and LPS, its main ligand ^[29]^. The TLR4 signaling pathway is considered to be one of the main triggers of the inflammatory response induced by obesity. TLR4 expression increases in response to HFD ^[2,25]^, and the resulting proinflammatory signaling in the intestine leads to the production of interleukin (IL)-1β, IL-6, tumor necrosis factor (TNF)-α, and interferon (IFN)-γ by local macrophages and T cells ^[27,30–32]^. Increased adiposity is associated with an increase in proinflammatory T cells and macrophages in the gut of humans ^[32]^ and mice ^[25]^, and along with increased infiltration of these cells in the gut, increased levels of proinflammatory cytokines were found in both species. In contrast, anti-inflammatory cells, such as regulatory T cells (Treg), and their products, such as IL-10, tend to decrease in the obese state ^[27,31]^. Treatment of mice on HFD with a gut-specific anti-inflammatory agent ameliorated many aspects of excessive adiposity, pointing to a key role for gut inflammation in the development of obesity and comorbidities such as IR ^[31]^. Together, these studies point to a subclinical level of inflammation that perpetuates the weakened gut barrier, endotoxemia, and systemic inflammation.

## 4. Metabolic inflammation and adiposity

Systemic inflammation activated by dysbiosis and a weakened gut barrier leads to inflammation in metabolic tissues (ie, metabolic inflammation). The most predominant tissue involved in metabolic inflammation is WAT. WAT is organized into several depots throughout the body, including multiple visceral and subcutaneous depots ^[33]^. Adipocytes were long characterized as only a fat storage location, but we now know they play an important role in metabolism as well as endocrine and immune function. They are capable of releasing microRNAs, lipids, and adipokines, the two most significant of the latter being lectin and adiponectin ^[34]^. Leptin controls satiety, and adiponectin regulates insulin sensitivity, and both have been studied in depth related to obesity. Leptin levels in the blood are directly proportional to the mass of WAT, and obesity leads to high levels of leptin in the systemic circulation. In addition to hormone production, adipocytes work in tandem with the stromal vascular faction (SVF), which contains a number of immune cells, including macrophages, B cells, and T cells ^[35]^, to maintain homeostasis in WAT.

During obesity, adipocytes, particularly in the visceral WAT depots, grow in size due to excess calorie consumption. The enlargement of adipocytes leads to changes in tissue structure and increased stress signaling (reviewed in ^[33]^), the latter of which triggers the production of proinflammatory cytokines and chemokines including, IL-1β, IL-6, IL-8, IL-12, macrophage chemoattractant protein-1, and TNF-α ^[36–38]^. Macrophages play a central role in the WAT inflammation seen in obesity. Macrophages of the SVF create so-called “crown-like structures” in WAT. The macrophages in these structures appear to play a role in lipid metabolism but can also promote WAT inflammation in obesity through proinflammatory signaling ^[39]^. The number of macrophages in WAT is positively correlated with adipocyte size and body mass, and these macrophages produce large amounts of IL-6 and TNF-α ^[37]^. TNF-α signaling in WAT has been shown to directly block insulin signaling ^[38]^. Other innate immune cells, including mast cells ^[40]^, innate lymphoid cells (reviewed in ^[41]^), and natural killer cells ^[42]^, also appear to play a role in WAT inflammation during obesity.

Adaptive immune cells also play a critical role in the development of WAT inflammation during adiposity. Cytotoxic T cells accumulate in the visceral WAT of mice fed HFD and contribute to inflammation, macrophage recruitment, and IR. T Helper (T_H_) cells coordinate many of the inflammatory processes during obesity. The number of T cell receptor αβ+ T cells in WAT correlates in a positive fashion with WAT mass ^[42]^. T_H_ cells guide the differentiation of WAT macrophage populations through cytokine production ^[43,44]^. The direction of changes to specific T_H_ subsets in WAT during obesity is less clear and seems to differ between models and experiments. For example, T_H_1 cells tend to be positively associated with adiposity ^[44,45]^, while T_H_2 cells have been shown to increase ^[46]^ or decrease ^[43]^, depending on the experimental conditions and timing of observations. Cytokines from T_H_2 cells can polarize macrophages toward the more anti-inflammatory (alternatively activated) phenotype. Treg, potent anti-inflammatory cells, are generally associated with the lean phenotype ^[45]^ but have been shown to increase in obesity, most likely as a compensatory mechanism to metabolic inflammation ^[46]^.

WAT inflammation contributes to IR, which can lead to T2DM. IR is due to endocrine effects, not only in WAT but in other highly metabolically active tissues such as skeletal muscle and liver. Although not as well studied as WAT, skeletal muscle does experience some level of inflammation during obesity and contributes to metabolic inflammation through the secretion of myokines (reviewed in ^[47]^). This inflammation is due to increased numbers of macrophages and T cells entering the tissue and the release of proinflammatory cytokines, such as IL-6 and TNF-α. Macrophages and their production of TNF-α, in particular, contribute to atrophy (ie, sarcopenic obesity) seen in many cases of obesity ^[48,49]^. Proinflammatory cytokines and other factors released by hypertrophic WAT can lead to atrophy and inflammation, subsequent to the production of IL-1β and IL-6 in skeletal muscle ^[50,51]^. The deposition of lipids in muscle ^[52–54]^ along with proinflammatory cytokine production ^[55,56]^ contributes to whole-body IR. As with the other tissues discussed thus far, anti-inflammatory IL-10 can reverse or block these negative effects ^[55,57]^. Skeletal muscle is responsible for 80% of glucose absorption under normal conditions; thus, resistance to insulin signaling in this tissue can greatly affect the function of skeletal muscle and promote IR and T2DM systemically ^[47,58]^. The effects of obesity are not limited to skeletal muscle. Smooth muscle myocytes secrete many cytokines and other molecules, such as IL-6, TNF-α, and myonectin, related to the inflammatory process and can become inflamed in obesity, leading to increased muscle inflammation ^[47]^.

Obesity is strongly associated with nonalcoholic fatty liver disease (NAFLD), where the accumulation of ectopic fat causes injury and inflammation to the liver. IR causes triglycerides to build up in hepatocytes, which contributes to NAFLD and increases the potential to develop cirrhosis and/or hepatocellular carcinoma ^[59]^. Cytotoxic T cells and macrophages accumulate in the liver during NAFLD, leading to chronic inflammation ^[60]^. Additionally, levels of inflammatory cytokines, including IL-6, IL-12, IFN-γ, and TNF-α, increase in the liver of obese mice regardless of disease state ^[61,62]^. Increases in TNF-α (and other cytokines that promote TNF-α) promote LPS-induced liver injury ^[62]^. TNF-α is increased in NAFLD and has been found to mediate the early stages of NAFLD as well as the transition to end-stage liver disease ^[59,63]^. The production of TNF-α early in the disease induces the production of other cytokines, such as IL-6, in the area, causing increased inflammation and infiltration of immune cells ^[63]^. IL-6 is markedly increased in patients with severe NAFLD compared with healthy patients ^[64]^ and contributes to the development of obesity-related tumors ^[61]^. Furthermore, IL-6 alters the liver’s response to insulin, leading to IR ^[55]^. The chronic inflammation in the liver in obese subjects leads to fibrosis and eventually cirrhosis ^[2,60,62,65]^. The eventual loss of liver function leads to significant health impacts.

## 5. Summary

The etiology of adiposity and obesity involves many factors, principle among these are dysbiosis and metabolic inflammation. Thus, manipulations of these physiological parameters have long been suggested as potential treatments for obesity. The use of prebiotics ^[66,67]^ and probiotics ^[23,25,68,69]^ to stabilize the gut microbiota has demonstrated reasonable success in ameliorating or preventing adiposity and certain comorbidities. In humans, the use of probiotics as a treatment for obesity and metabolic disease has shown positive results, although higher doses and longer duration of treatment (compared with rodent models) seem necessary for the best effects ^[68,70–72]^. Probiotics are likely not a “silver bullet”, but can be an effective complementary approach to other treatments ^[72]^. Similarly, the use of anti-inflammatory drugs, either systemic ^[73–75]^ or gut-specific ^[31]^, can also limit fattening, IR, and other effects of metabolic inflammation in laboratory models. Clinical studies in humans are less abundant but do reveal some positive anti-inflammatory effects in WAT ^[75]^. It is possible that a combination of these treatments may be more effective in treating adiposity and metabolic disease.

Many inbred laboratory rodent models of obesity exist, including diet-induced, monogenic, and polygenic models ^[76]^. Because of the heterogeneity of obesity in humans, outbred models have been suggested as a better laboratory model for human disease ^[77]^. Hibernating mammals, such as ground squirrels, represent a natural and outbred model for rapid adiposity and IR. These animals undergo a yearly cycle of fattening (preparing for hibernation) and weight loss (during hibernation), even on a calorie-restricted diet ^[78]^. Hibernators also develop IR late in the active season only to become insulin sensitive again at the onset of hibernation ^[79]^. Interestingly, the development of adiposity in ground squirrels mirrors the metabolic inflammation seen in obese rodent models and humans ^[80]^. Thus, these animals serve as a good model in which to test the effects of manipulations of gut microbiota and inflammation as a treatment for adiposity and obesity. Hopefully, the use of outbred models more similar to humans in their heterogeneity of exposure, microbiota, and physiology can lead to better treatments for obesity that focus on curbing dysbiosis and metabolic inflammation.

Obesity is a heterogeneous disease, and treatments must be tailored to the individual patient. The pathway that we describe here represents a common etiology that arises from a changed diet and leads to the metabolic inflammation that is present in most cases of the disease. Changes to the gut microbiota and activation of the immune system are involved in many other diseases as well, including neurological disorders (reviewed in ^[81–83]^). It is important to develop a deeper understanding of the interactions between these important aspects of physiology to develop better treatments for a wide range of diseases in the future.

## Conflicts of interest

The authors declare no conflicts of interest.

## Funding

This work was supported by the National Institutes of Health award (2R15GM124586-02—“Microbiota and Inflammation in Adiposity: The Ground Squirrel Model”).

## References

[1] Woods SC. The control of food intake: behavioral versus molecular perspectives. Cell Metab. 2009;9(6):489–98. 19490904 10.1016/j.cmet.2009.04.007 PMC3090647

[2] Suriano F Vieira-Silva S Falony G. Fat and not sugar as the determining factor for gut microbiota changes, obesity, and related metabolic disorders in mice. Am J Physiol Endocrinol Metab. 2023;324(1):E85–96. 36516223 10.1152/ajpendo.00141.2022

[3] Agagunduz D Icer MA Yesildemir O. The roles of dietary lipids and lipidomics in gut-brain axis in type 2 diabetes mellitus. J Transl Med. 2023;21(1):240. 37009872 10.1186/s12967-023-04088-5 PMC10068184

[4] Agus A Clement K Sokol H. Gut microbiota-derived metabolites as central regulators in metabolic disorders. Gut. 2021;70(6):1174–82. 33272977 10.1136/gutjnl-2020-323071 PMC8108286

[5] Allam-Ndoul B Castonguay-Paradis S Veilleux A. Gut microbiota and intestinal trans-epithelial permeability. Int J Mol Sci . 2020;21(17):6402. 32899147 10.3390/ijms21176402 PMC7503654

[6] Forgie AJ Fouhse JM Willing BP. Diet-microbe-host interactions that affect gut mucosal integrity and infection resistance. Front Immunol. 2019;10:1802. 31447837 10.3389/fimmu.2019.01802 PMC6691341

[7] Scheithauer TPM Rampanelli E Nieuwdorp M. Gut microbiota as a trigger for metabolic inflammation in obesity and type 2 diabetes. Front Immunol. 2020;11:571731. 33178196 10.3389/fimmu.2020.571731 PMC7596417

[8] Chelakkot C Choi Y Kim DK. Akkermansia muciniphila-derived extracellular vesicles influence gut permeability through the regulation of tight junctions. Exp Mol Med. 2018;50(2):e450. 29472701 10.1038/emm.2017.282 PMC5903829

[9] Moreira AP Texeira TF Ferreira AB. Influence of a high-fat diet on gut microbiota, intestinal permeability and metabolic endotoxaemia. Br J Nutr. 2012;108(5):801–9. 22717075 10.1017/S0007114512001213

[10] Guo X Li S Zhang J. Genome sequencing of 39 Akkermansia muciniphila isolates reveals its population structure, genomic and functional diverisity, and global distribution in mammalian gut microbiotas. BMC Genomics. 2017;18(1):800. 29047329 10.1186/s12864-017-4195-3 PMC5648452

[11] Zhai Q Feng S Arjan N. A next generation probiotic, Akkermansia muciniphila. Crit Rev Food Sci Nutr. 2019;59(19):3227–36. 30373382 10.1080/10408398.2018.1517725

[12] Parada Venegas D De la Fuente MK Landskron G. Short Chain Fatty Acids (SCFAs)-mediated gut epithelial and immune regulation and its relevance for inflammatory bowel diseases. Front Immunol. 2019;10:277. 30915065 10.3389/fimmu.2019.00277 PMC6421268

[13] Lee EY Bang JY Park GW. Global proteomic profiling of native outer membrane vesicles derived from Escherichia coli. Proteomics. 2007;7(17):3143–53. 17787032 10.1002/pmic.200700196

[14] Horstman AL Kuehn MJ. Bacterial surface association of heat-labile enterotoxin through lipopolysaccharide after secretion via the general secretory pathway. J Biol Chem. 2002;277(36):32538–45. 12087095 10.1074/jbc.M203740200 PMC4391702

[15] Kuehn MJ Kesty NC. Bacterial outer membrane vesicles and the host-pathogen interaction. Genes Dev. 2005;19(22):2645–55. 16291643 10.1101/gad.1299905

[16] Le CE Nielsen T Qin J. Richness of human gut microbiome correlates with metabolic markers. Nature. 2013;500(7464):541–6. 23985870 10.1038/nature12506

[17] Turnbaugh PJ Hamady M Yatsunenko T. A core gut microbiome in obese and lean twins. Nature. 2009;457(7228):480–4. 19043404 10.1038/nature07540 PMC2677729

[18] Pinart M Dotsch A Schlicht K. Gut microbiome composition in obese and non-obese persons: a systematic review and meta-analysis. Nutrients. 2021;14(1):12. 35010887 10.3390/nu14010012 PMC8746372

[19] Ley RE Backhed F Turnbaugh P. Obesity alters gut microbial ecology. Proc Natl Acad Sci U S A. 2005;102(31):11070–5. 16033867 10.1073/pnas.0504978102 PMC1176910

[20] Pascale A Marchesi N Marelli C. Microbiota and metabolic diseases. Endocrine. 2018;61(3):357–71. 29721802 10.1007/s12020-018-1605-5

[21] Hildebrandt MA Hoffmann C Sherrill-Mix SA. High-fat diet determines the composition of the murine gut microbiome independently of obesity. Gastroenterology. 2009;137(5):1716–24.e1. 19706296 10.1053/j.gastro.2009.08.042 PMC2770164

[22] Lam YY Ha CW Campbell CR. Increased gut permeability and microbiota change associate with mesenteric fat inflammation and metabolic dysfunction in diet-induced obese mice. PLoS One. 2012;7(3):e34233. 22457829 10.1371/journal.pone.0034233 PMC3311621

[23] Kang Y Kang X Yang H. Lactobacillus acidophilus ameliorates obesity in mice through modulation of gut microbiota dysbiosis and intestinal permeability. Pharmacol Res. 2022;175:106020. 34896249 10.1016/j.phrs.2021.106020

[24] Cani PD Possemiers S Van de Wiele T. Changes in gut microbiota control inflammation in obese mice through a mechanism involving GLP-2-driven improvement of gut permeability. Gut. 2009;58(8):1091–103. 19240062 10.1136/gut.2008.165886 PMC2702831

[25] Moya-Perez A Neef A Sanz Y. Bifidobacterium pseudocatenulatum CECT 7765 reduces obesity-associated inflammation by restoring the lymphocyte-macrophage balance and gut microbiota structure in high-fat diet-fed mice. PLoS One. 2015;10(7):e0126976. 26161548 10.1371/journal.pone.0126976 PMC4498624

[26] Cox AJ West NP Cripps AW. Obesity, inflammation, and the gut microbiota. Lancet Diabetes Endocrinol. 2015;3(3):207–15. 25066177 10.1016/S2213-8587(14)70134-2

[27] Hamilton MK Boudry G Lemay DG. Changes in intestinal barrier function and gut microbiota in high-fat diet-fed rats are dynamic and region dependent. Am J Physiol Gastrointest Liver Physiol. 2015;308(10):G840–51. 25747351 10.1152/ajpgi.00029.2015 PMC4437018

[28] Mogensen TH. Pathogen recognition and inflammatory signaling in innate immune defenses. Clin Microbiol Rev. 2009;22(2):240–73, Table of Contents. 19366914 10.1128/CMR.00046-08 PMC2668232

[29] Taniguchi Y Yoshioka N Nakata K. Mechanism for maintaining homeostasis in the immune system of the intestine. Anticancer Res. 2009;29(11):4855–60. 20032447

[30] Liu W Crott JW Lyu L. Diet- and genetically-induced obesity produces alterations in the microbiome, inflammation and Wnt pathway in the intestine of Apc(+/1638N) mice: comparisons and contrasts. J Cancer. 2016;7(13):1780–90. 27698916 10.7150/jca.15792 PMC5039360

[31] Luck H Tsai S Chung J. Regulation of obesity-related insulin resistance with gut anti-inflammatory agents. Cell Metab. 2015;21(4):527–42. 25863246 10.1016/j.cmet.2015.03.001

[32] Rohm TV Fuchs R Muller RL. Obesity in humans is characterized by gut inflammation as shown by pro-inflammatory intestinal macrophage accumulation. Front Immunol. 2021;12:668654. 34054838 10.3389/fimmu.2021.668654 PMC8158297

[33] Kahn CR Wang G Lee KY. Altered adipose tissue and adipocyte function in the pathogenesis of metabolic syndrome. J Clin Invest. 2019;129(10):3990–4000. 31573548 10.1172/JCI129187 PMC6763230

[34] Ouchi N Walsh K. Adiponectin as an anti-inflammatory factor. Clin Chim Acta. 2007;380(1-2):24–30. 17343838 10.1016/j.cca.2007.01.026 PMC2755046

[35] Blaszczak AM Jalilvand A Hsueh WA. Adipocytes, innate immunity and obesity: a mini-review. Front Immunol. 2021;12:650768. 34248937 10.3389/fimmu.2021.650768 PMC8264354

[36] Kunz HE Hart CR Gries KJ. Adipose tissue macrophage populations and inflammation are associated with systemic inflammation and insulin resistance in obesity. Am J Physiol Endocrinol Metab. 2021;321(1):E105–21. 33998291 10.1152/ajpendo.00070.2021 PMC8321823

[37] Weisberg SP McCann D Desai M. Obesity is associated with macrophage accumulation in adipose tissue. J Clin Invest. 2003;112(12):1796–808. 14679176 10.1172/JCI19246 PMC296995

[38] Hotamisligil GS Shargill NS Spiegelman BM. Adipose expression of tumor necrosis factor-alpha: direct role in obesity-linked insulin resistance. Science. 1993;259(5091):87–91. 7678183 10.1126/science.7678183

[39] Hill DA Lim HW Kim YH. Distinct macrophage populations direct inflammatory versus physiological changes in adipose tissue. Proc Natl Acad Sci U S A. 2018;115(22):E5096–105. 29760084 10.1073/pnas.1802611115 PMC5984532

[40] Lopez-Perez D Redruello-Romero A Garcia-Rubio J. In patients with obesity, the number of adipose tissue mast cells is significantly lower in subjects with type 2 diabetes. Front Immunol. 2021;12:664576. 34093556 10.3389/fimmu.2021.664576 PMC8177010

[41] LaMarche NM Kohlgruber AC Brenner MB. Innate T cells govern adipose tissue biology. J Immunol. 2018;201(7):1827–34. 30224362 10.4049/jimmunol.1800556 PMC6201318

[42] Caspar-Bauguil S Cousin B Andre M. Weight-dependent changes of immune system in adipose tissue: importance of leptin. Exp Cell Res. 2006;312(12):2195–202. 16650847 10.1016/j.yexcr.2006.03.023

[43] Brinker G Froeba J Arndt L. CD4(+) T cells regulate glucose homeostasis independent of adipose tissue dysfunction in mice. Eur J Immunol. 2021;51(6):1399–411. 33784418 10.1002/eji.202048870

[44] Strissel KJ DeFuria J Shaul ME. T-cell recruitment and Th1 polarization in adipose tissue during diet-induced obesity in C57BL/6 mice. Obesity (Silver Spring). 2010;18(10):1918–25. 20111012 10.1038/oby.2010.1 PMC2894258

[45] Feuerer M Herrero L Cipolletta D. Lean, but not obese, fat is enriched for a unique population of regulatory T cells that affect metabolic parameters. Nat Med. 2009;15(8):930–9. 19633656 10.1038/nm.2002 PMC3115752

[46] Zeyda M Huber J Prager G. Inflammation correlates with markers of T-cell subsets including regulatory T cells in adipose tissue from obese patients. Obesity (Silver Spring). 2011;19(4):743–8. 20508627 10.1038/oby.2010.123

[47] Wu H Ballantyne CM. Skeletal muscle inflammation and insulin resistance in obesity. J Clin Invest. 2017;127(1):43–54. 28045398 10.1172/JCI88880 PMC5199705

[48] Jackman RW Kandarian SC. The molecular basis of skeletal muscle atrophy. Am J Physiol Cell Physiol. 2004;287(4):C834–43. 15355854 10.1152/ajpcell.00579.2003

[49] Kalinkovich A Livshits G. Sarcopenic obesity or obese sarcopenia: a cross talk between age-associated adipose tissue and skeletal muscle inflammation as a main mechanism of the pathogenesis. Ageing Res Rev. 2017;35:200–21. 27702700 10.1016/j.arr.2016.09.008

[50] Pellegrinelli V Rouault C Rodriguez-Cuenca S. Human adipocytes induce inflammation and atrophy in muscle cells during obesity. Diabetes. 2015;64(9):3121–34. 25695947 10.2337/db14-0796

[51] Zhu S Tian Z Torigoe D. Aging- and obesity-related peri-muscular adipose tissue accelerates muscle atrophy. PLoS One. 2019;14(8):e0221366. 31442231 10.1371/journal.pone.0221366 PMC6707561

[52] Virkamaki A Korsheninnikova E Seppala-Lindroos A. Intramyocellular lipid is associated with resistance to in vivo insulin actions on glucose uptake, antilipolysis, and early insulin signaling pathways in human skeletal muscle. Diabetes. 2001;50(10):2337–43. 11574417 10.2337/diabetes.50.10.2337

[53] Krssak M Falk Petersen K Dresner A. Intramyocellular lipid concentrations are correlated with insulin sensitivity in humans: a 1H NMR spectroscopy study. Diabetologia. 1999;42(1):113–6. 10027589 10.1007/s001250051123

[54] Perseghin G Scifo P De Cobelli F. Intramyocellular triglyceride content is a determinant of in vivo insulin resistance in humans: a 1H-13C nuclear magnetic resonance spectroscopy assessment in offspring of type 2 diabetic parents. Diabetes. 1999;48(8):1600–6. 10426379 10.2337/diabetes.48.8.1600

[55] Kim HJ Higashimori T Park SY. Differential effects of interleukin-6 and -10 on skeletal muscle and liver insulin action in vivo. Diabetes. 2004;53(4):1060–7. 15047622 10.2337/diabetes.53.4.1060

[56] Borst SE Lee Y Conover CF. Neutralization of tumor necrosis factor-alpha reverses insulin resistance in skeletal muscle but not adipose tissue. Am J Physiol Endocrinol Metab. 2004;287(5):E934–8. 15213061 10.1152/ajpendo.00054.2004

[57] Hong EG Ko HJ Cho YR. Interleukin-10 prevents diet-induced insulin resistance by attenuating macrophage and cytokine response in skeletal muscle. Diabetes. 2009;58(11):2525–35. 19690064 10.2337/db08-1261 PMC2768157

[58] DeFronzo RA Tripathy D. Skeletal muscle insulin resistance is the primary defect in type 2 diabetes. Diabetes Care. 2009;32:S157–63. 19875544 10.2337/dc09-S302 PMC2811436

[59] Assuncao SNF Sorte N Alves CAD. Inflammatory cytokines and non-alcoholic fatty liver disease (NAFLD) in obese children and adolescents. Nutr Hosp. 2018;35(1):78–83. 29565153 10.20960/nh.1317

[60] Breuer DA Pacheco MC Washington MK. CD8(+) T cells regulate liver injury in obesity-related nonalcoholic fatty liver disease. Am J Physiol Gastrointest Liver Physiol. 2020;318(2):G211–24. 31709830 10.1152/ajpgi.00040.2019 PMC7052570

[61] Park EJ Lee JH Yu GY. Dietary and genetic obesity promote liver inflammation and tumorigenesis by enhancing IL-6 and TNF expression. Cell. 2010;140(2):197–208. 20141834 10.1016/j.cell.2009.12.052 PMC2836922

[62] Yang SQ Lin HZ Lane MD. Obesity increases sensitivity to endotoxin liver injury: implications for the pathogenesis of steatohepatitis. Proc Natl Acad Sci U S A. 1997;94(6):2557–62. 9122234 10.1073/pnas.94.6.2557 PMC20127

[63] Tilg H Diehl AM. Cytokines in alcoholic and nonalcoholic steatohepatitis. N Engl J Med. 2000;343(20):1467–76. 11078773 10.1056/NEJM200011163432007

[64] Wieckowska A Papouchado BG Li Z. Increased hepatic and circulating interleukin-6 levels in human nonalcoholic steatohepatitis. Am J Gastroenterol. 2008;103(6):1372–9. 18510618 10.1111/j.1572-0241.2007.01774.x

[65] Salles J Tardif N Landrier JF. TNFalpha gene knockout differentially affects lipid deposition in liver and skeletal muscle of high-fat-diet mice. J Nutr Biochem. 2012;23(12):1685–93. 22464148 10.1016/j.jnutbio.2011.12.001

[66] Neyrinck AM Van Hee VF Piront N. Wheat-derived arabinoxylan oligosaccharides with prebiotic effect increase satietogenic gut peptides and reduce metabolic endotoxemia in diet-induced obese mice. Nutr Diabetes. 2012;2:e28. 23154683 10.1038/nutd.2011.24 PMC3302144

[67] Jiang T Gao X Wu C. Apple-derived pectin modulates gut microbiota, improves gut barrier function, and attenuates metabolic endotoxemia in rats with diet-induced obesity. Nutrients. 2016;8(3):126. 26938554 10.3390/nu8030126 PMC4808856

[68] Depommier C Everard A Druart C. Supplementation with Akkermansia muciniphila in overweight and obese human volunteers: a proof-of-concept exploratory study. Nat Med. 2019;25(7):1096–103. 31263284 10.1038/s41591-019-0495-2 PMC6699990

[69] Plovier H Everard A Druart C. A purified membrane protein from Akkermansia muciniphila or the pasteurized bacterium improves metabolism in obese and diabetic mice. Nat Med. 2017;23(1):107–13. 27892954 10.1038/nm.4236

[70] Kopp L Schweinlin A Tingo L. Potential modulation of inflammation and physical function by combined probiotics, omega-3 supplementation and vitamin D supplementation in overweight/obese patients with chronic low-grade inflammation: a randomized, placebo-controlled trial. Int J Mol Sci . 2023;24(10):8567. 37239916 10.3390/ijms24108567 PMC10217964

[71] Guedes MR Pontes K Barreto Silva MI. Randomized controlled trials reporting the effects of probiotics in individuals with overweight and obesity: a critical review of the interventions and body adiposity parameters. Clin Nutr. 2023;42(6):835–47. 37084470 10.1016/j.clnu.2023.03.017

[72] Soltani S Ashoori M Dehghani F. Effects of probiotic/synbiotic supplementation on body weight in patients with diabetes: a systematic review and meta-analyses of randomized-controlled trials. BMC Endocr Disord. 2023;23(1):86. 37085813 10.1186/s12902-023-01338-x PMC10120130

[73] Kim JK Kim YJ Fillmore JJ. Prevention of fat-induced insulin resistance by salicylate. J Clin Invest. 2001;108(3):437–46. 11489937 10.1172/JCI11559 PMC209353

[74] Yuan M Konstantopoulos N Lee J. Reversal of obesity- and diet-induced insulin resistance with salicylates or targeted disruption of Ikkbeta. Science. 2001;293(5535):1673–7. 11533494 10.1126/science.1061620

[75] Nixon M Wake DJ Livingstone DE. Salicylate downregulates 11beta-HSD1 expression in adipose tissue in obese mice and in humans, mediating insulin sensitization. Diabetes. 2012;61(4):790–6. 22357964 10.2337/db11-0931 PMC3314355

[76] Nilsson C Raun K Yan FF. Laboratory animals as surrogate models of human obesity. Acta Pharmacol Sin. 2012;33(2):173–81. 22301857 10.1038/aps.2011.203 PMC4010334

[77] Gordon-Larsen P French JE Moustaid-Moussa N. Synergizing mouse and human studies to understand the heterogeneity of obesity. Adv Nutr. 2021;12(5):2023–34. 33885739 10.1093/advances/nmab040 PMC8483969

[78] Hatton JJ Stevenson TJ Buck CL. Diet affects arctic ground squirrel gut microbial metatranscriptome independent of community structure. Environ Microbiol. 2017;19(4):1518–35. 28251799 10.1111/1462-2920.13712 PMC5417852

[79] Florant GL Lawrence AK Williams K. Seasonal changes in pancreatic B-cell function in euthermic yellow-bellied marmots. Am J Physiol. 1985;249(2 Pt 2):R159–65. 3895984 10.1152/ajpregu.1985.249.2.R159

[80] Sonsalla MM Love SL Hoh LJ. Development of metabolic inflammation during pre-hibernation fattening in 13-lined ground squirrels (Ictidomys tridecemlineatus). J Comp Physiol B. 2021;191(5):941–53. 34165591 10.1007/s00360-021-01384-8

[81] Agagunduz D Gencer Bingol F Celik E. Recent developments in the probiotics as live biotherapeutic products (LBPs) as modulators of gut brain axis related neurological conditions. J Transl Med. 2022;20(1):460. 36209124 10.1186/s12967-022-03609-y PMC9548122

[82] Agagunduz D Kocaadam-Bozkurt B Bozkurt O. Microbiota alteration and modulation in Alzheimer’s disease by gerobiotics: The gut-health axis for a good mind. Biomed Pharmacother. 2022;153:113430. 36076486 10.1016/j.biopha.2022.113430

[83] Ortega MA Alvarez-Mon MA Garcia-Montero C. Microbiota-gut-brain axis mechanisms in the complex network of bipolar disorders: potential clinical implications and translational opportunities. Mol Psychiatry. 2023. doi: 10.1038/s41380-023-01964-w. 10.1038/s41380-023-01964-w PMC10615769 36707651

